# *Lactiplantibacillus plantarum* BF_15 Confers Protection Against Ulcerative Colitis by Orchestrating Gut Microbiota‐Barrier‐Immune Triad Homeostasis

**DOI:** 10.1002/fsn3.72275

**Published:** 2026-09-04

**Authors:** Na Zhang, Hongjie Liu, Dongyao Li, Chen Li, Xinyi Ge, Hongyan Kang, Miaoshu Wang, Hongtao Tian, Xiaoyan Miao

**Affiliations:** ^1^ College of Food Science and Technology Hebei Agricultural University Baoding Hebei China; ^2^ College of Biochemistry and Environmental Engineering Baoding University Baoding Hebei China; ^3^ National Engineering Research Center for Agriculture in Northern Mountainous Areas Baoding Hebei China; ^4^ New Hope Tensun (Hebei) Dairy Co, Ltd Baoding Hebei China

**Keywords:** gut microbiota, *Lactiplantibacillus plantarum*, proteomics, the antigen presentation and processing (APC) pathway, ulcerative colitis

## Abstract

Ulcerative colitis (UC) is a prevalent chronic inflammatory bowel disease globally, driven mainly by immune dysregulation and gut microbiota dysbiosis. This study focused on *Lactiplantibacillus plantarum* BF_15, an infant‐derived strain with potential to alleviate UC. Four experimental groups were established, including the control group, DSS group, BF_15 group, and LGG group. Probiotics were administrated at a dosage of 1 × 10^9^ CFU/mL and 0.1 mL/10 g body weight (BW) for 21 days. The therapeutic effects and mechanisms of BF_15 against UC were explored based on physiological indicators, gut microbiota, and tandem mass tag (TMT)‐based quantitative proteomics. The results showed that BF_15 intervention significantly reduced the disease activity index (DAI) from 3.00 ± 0.50 to 1.16 ± 0.36 (*p* < 0.05) and mitigated UC‐related symptoms including body weight loss and colon shortening, with colon length restored from 4.43 ± 0.32 cm to 5.41 ± 0.26 cm (*p* < 0.05). Meanwhile, BF_15 downregulated the levels of pro‐inflammatory factors such as interleukin IL‐6 and IL‐17A, and upregulated the levels of IL‐10 (from 565.92 ± 79.40 pg/mL to 656.84 ± 66.01 pg/mL) and secretory immunoglobulin A (SIgA, from 9.75 ± 1.29 μg/mL to 12.79 ± 1.77 μg/mL) (*p* < 0.05), with a therapeutic effect comparable to that of LGG. Further, gut microbiota analysis demonstrated that BF_15 restored the relative abundance of *Bacteroidetes* (from 28.02% to 30.85%) and regulated gut microbiota homeostasis. Finally, TMT‐based proteomic analysis indicated that BF_15 regulated 528 differentially expressed proteins (DEPs) and exhibited a superior regulatory effect on the antigen presentation and processing (APC) pathway compared with LGG. The above findings provide potential novel therapeutic targets and probiotic strain resources for the treatment of UC.

## Graphical abstract

*Lactiplantibacillus plantarum* BF_15 mitigates ulcerative colitis via modulating the triad homeostasis of gut microbiota, mucosal barrier and intestinal immunity. It restores disturbed intestinal microecology by elevating the abundance of *Bacteroidetes*. Modulation of the antigen presentation and processing (APC) pathway suppresses antigen‐presenting cell overactivation, reduces pro‐inflammatory TNF‐α and IL‐6 production, and enhances anti‐inflammatory IL‐10 secretion. BF_15 also boosts intestinal SIgA release to strengthen mucosal barrier function. Taken together, these regulatory actions reverse body weight loss and colon shortening in colitic mice and elicit stable colonic protective effects.

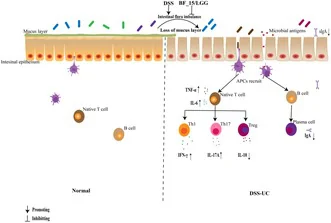

## 1 Introduction

Ulcerative colitis (UC) is an inflammatory bowel disease characterized by persistent colonic inflammation and mucosal ulceration (Guo et al. 2024). It has become a prominent global public health challenge, seriously endangering patients' health and reducing their quality of life (Wang et al. 2019). Currently, long‐term use of first‐line therapeutic drugs for UC (5‐aminosalicylic acids, glucocorticoids, and immunosuppressants) is prone to cause adverse effects, increase disease recurrence rate, and reduce patient tolerance, prompting researchers to explore safer and more effective alternative therapeutic strategies (Parikh et al. 2019). A large number of studies have shown that gut microbiota dysbiosis is a key initiating factor of UC, which can damage the intestinal mucosal barrier, leading to the translocation of pathogen‐associated molecular patterns (PAMPs) and excessive release of pro‐inflammatory cytokines (such as IL‐1β and IL‐6), thereby exacerbating colonic inflammation (Dai et al. 2023). Persistent inflammation, in turn, further disrupts the intestinal flora and impairs the barrier function, forming a vicious cycle (Dai et al. 2023). Therefore, restoring intestinal microecological balance and reconstructing the intestinal mucosal barrier have become potential directions and important breakthroughs in the prevention and treatment of UC (Markowiak and Śliżewska 2017).

As live microorganisms that confer health benefits on the host, probiotics from diverse sources have been widely demonstrated to exert UC‐alleviating probiotic properties via multiple pathways, including regulating gut microbiota, improving host metabolism, enhancing intestinal mucosal barrier function, and modulating the immune system (Öhman and Simrén 2010; Zhang et al. 2024). *Ligilactobacillus pentosus* YQ001 and *Ligilactobacillus senioris* YQ005, isolated from fermented bamboo shoots, have been validated via in vitro screening and DSS‐induced UC mouse models to exhibit superior probiotic activity compared with the commonly used reference strain LGG. These strains can effectively ameliorate colonic pathological damage and relieve UC symptoms by restoring gut microbiota homeostasis, inhibiting the expression of pro‐inflammatory factors, and upregulating the levels of intestinal barrier‐related proteins including zonula occludens‐1 (ZO‐1), Occludin, and cathelicidin‐related antimicrobial peptide (CRAMP) (Liu et al. 2024). Orally administered *L. plantarum* HY7718, isolated from fermented squid, has been shown to significantly alleviate symptoms and improve intestinal integrity in DSS‐induced colitis mice. This effect is mediated by upregulating the expression of intestinal tight junction (TJ)‐related genes, downregulating the expression of pro‐inflammatory cytokines and genes associated with the Toll‐like receptor (TLR)/myeloid differentiation factor 88 (MyD88)/nuclear factor kappa‐B (NF‐κB) signaling pathway, and modulating gut microbiota composition (Kim et al. 2024). *Lactobacillus helveticus* LZ‐R‐5, isolated from traditional fermented kefir grains in Linzhi, Tibet, exhibits promising UC‐alleviating potential. It can ameliorate structural disorder of proximal colonic crypts and enhance intestinal barrier function by regulating the expression of transforming growth factor‐β1 (TGF‐β1), NOD‐like receptor thermal protein domain associated protein 3 (NLRP3)‐related inflammatory factors, and G protein‐coupled receptor 43 (GPR43), as well as modulating gut microbiota composition (Zhao et al. 2025). Compared with probiotics derived from traditional fermented products, human‐derived probiotics (particularly mother‐infant‐derived probiotics) have more prominent advantages in UC prevention and treatment due to their superior human biocompatibility, safety, and functional adaptability. Previous studies have demonstrated that *Bifidobacterium breve* CCFM683, isolated from the intestinal tract of newborns, can effectively ameliorate DSS‐induced colonic tissue damage by downregulating pro‐inflammatory cytokines, upregulating anti‐inflammatory factors, and reshaping gut microbiota structure (Yang et al. 2018). At present, systematic studies on the anti‐UC effects of human‐derived/mother‐infant‐derived probiotics remain scarce, and their specific mechanisms of action have not been fully elucidated. This study focuses on infant‐derived probiotics from breastfed infants, a superior source of candidate strains, and conducts a systematic investigation to fill this research gap, with the aim of providing novel insights and candidate strains for the probiotic‐based prevention and treatment of UC.

*Lactiplantibacillus plantarum* BF_15 is a strain isolated from a healthy breastfed infant, with superior human biocompatibility compared with food‐derived strains. It can efficiently colonize the intestinal tract, and possesses well‐validated probiotic properties including immunomodulatory activity and oxidative stress alleviation (Zhang et al. 2020). The whole‐genome sequencing (WGS) of this strain has been completed, with the complete genome sequence deposited in NCBI GenBank under the accession numbers CP089516‐CP089517. Its relevant functional properties are protected by a granted Chinese invention patent (No. ZL202010193432.4), supporting its well‐characterized genetic background and reliable biosafety (Wang et al. 2022). Parallel animal trials were conducted using mice of the identical strain and batch as those adopted in the present study. The results verified that BF_15 markedly relieved intestinal mucosal erosion and inflammatory cell infiltration in DSS‐induced colitis mice. This strain significantly elevated colonic SOD activity and reduced MDA accumulation, further alleviating DSS‐triggered intestinal mucosal injury and systemic oxidative stress. No statistically significant difference in colon‐protective efficacy was observed between BF_15 and the positive control LGG. These preliminary data have been published in *Journal of Chinese Institute of Food Science and Technology* (Zhang et al. 2022). Taken together, *L. plantarum* BF_15 exhibits well‐documented probiotic potential for the amelioration of UC.

Based on our previous findings, this study further systematically explored the alleviating effect of *L. plantarum* BF_15 on DSS‐induced colitis in mice. High‐throughput 16S rRNA sequencing was applied to analyze its regulatory role in gut microbiota composition, and TMT‐based quantitative proteomics was further used to elucidate the underlying regulatory mechanisms of BF_15. This work aimed to provide novel theoretical support and experimental evidence for the probiotic intervention of UC.

## 2 Materials and Methods

### 2.1 Bacterial Strains

*L. plantarum* BF_15 was isolated from the fecal samples of exclusively breastfed infants and preserved in the culture collection of our laboratory. Whole‐genome sequencing (WGS) analysis was performed on this strain, with its complete genome sequence deposited in NCBI GenBank under the accession numbers CP089516‐CP089517. Following alignment against the Virulence Factor Database (VFDB), 116 virulence‐associated genes were identified in the BF_15 genome, most of which are defensive virulence genes. Only 3 invasive virulence genes were detected, which are essential for bacterial survival and also present in the commercially available food‐grade safe probiotic strain ST‐III. Alignment against the Comprehensive Antibiotic Resistance Database (CARD) revealed 94 antibiotic resistance genes, all of which are chromosomally encoded with no risk of horizontal gene transfer (HGT). Collectively, these genomic data confirm the biosafety of this strain, supporting its eligibility as a candidate preventive probiotic (Wang et al. 2022). LGG (ATCC 53103) was purchased from the China General Microbiological Culture Collection Center (CGMCC). The strains were activated and subcultured for 3 consecutive passages in de Man, Rogosa and Sharpe (MRS) broth; then the bacterial cells were harvested by centrifugation at 6000 rpm for 10 min, washed twice with sterile 0.9% normal saline, and resuspended to a final concentration of (1–2) × 10^9^ CFU/mL for intragastric administration.

### 2.2 Animals

Male C57BL/6J mice aged 7 weeks with a body weight of 20 ± 2 g were purchased from SPF (Beijing) Biotechnology Co. Ltd. [Laboratory Animal Production License No.: SCXK (Jing) 2024‐0001]. All animal experimental protocols were approved by the Animal Ethics Committee of Hebei Agricultural University (Approval No.: SCXK 2019‐0030), and all experiments were performed in strict accordance with the ARRIVE guidelines (Kilkenny et al. 2010). During the experiment, mice were housed in individually ventilated cages (IVCs) under controlled environmental conditions: temperature (23 ± 2), °C, relative humidity 50% ± 5%, and a 12 h light/dark cycle, with free access to standard chow and water. The mental state, activity, diet, and body weight of the mice were monitored daily. Mice were immediately euthanized to minimize suffering when body weight loss exceeded 20% or severe discomfort was observed. No invasive procedures were performed during the experiment, and mild distress was alleviated via environmental and nutritional intervention.

### 2.3 Experimental Design

Mice were housed under specific pathogen‐free (SPF) conditions in individually ventilated cages (IVCs) with a 12 h light/dark cycle, ambient temperature of 23°C ± 1°C, and relative humidity of 60% ± 5%. Animals had free access to autoclaved chow and water. After 7 days of acclimatization, mice were randomly divided into four groups (*n* = 10 per group):

- Control group: saline only;
- DSS group: saline + DSS;
- BF_15 group: BF_15 (1 × 10^9^ CFU/mL, 0.1 mL/10 g BW, daily);
- LGG group: LGG (1 × 10^9^ CFU/mL, 0.1 mL/10 g BW, daily).

The administration of probiotics (BF_15/LGG) via oral gavage was conducted for a duration of 3 weeks, with the dosage protocol referenced from Zhang et al. (2020). At Week 4, all experimental groups (excluding the control group) received 0.8 g/mL DSS solution (0.1 mL per 10 g body weight) by oral gavage for 7 days to establish an acute colitis model. Detailed procedural steps are provided in Figure S1.

The gavage‐based DSS administration was employed to mitigate experimental variability arising from inconsistent DSS intake in the free‐drinking model (Liang et al. 2025). Preliminary model optimization demonstrated that the gavage protocol (0.8 g/mL DSS, 0.1 mL/10 g body weight, 7 days) yielded superior uniformity of inflammatory indicators and reduced inter‐individual variability compared to the free‐drinking method (3.5% DSS for 7 days).

### 2.4 Disease Activity Index (DAI)

DAI scores were calculated according to Hamamoto et al. (1999), based on body weight change, stool consistency, and fecal occult blood (Table 1). Fecal occult blood was measured using a commercial kit (Zhuhai Besso Biotechnology Co. Ltd., Zhuhai, China).

**TABLE 1:** Scoring criteria for disease activity index (DAI).

Weight loss (%)	Stool consistency	Occult/gross bleeding	Score
0	Normal	Normal	0
1 ~ 5	Loose stools	Guiac+	1
5 ~ 10	—	—	2
10 ~ 15	Diarrhea	Gross bleeding	3
> 15	—	—	4

### 2.5 Sample Collection

At the end of the experiment, blood was collected from the orbital sinus under light anesthesia. Serum was separated by centrifugation (2000 rpm, 10 min) and stored at −80°C for cytokine analysis. Colons were excised from the anus to the ileocecal junction, measured for length, and visually inspected for gross pathology. Luminal contents were collected aseptically, and mucosal surfaces were rinsed with sterile saline. All samples were snap‐frozen in liquid nitrogen and stored at −80°C until further analysis.

### 2.6 Cytokine and SIgA Measurement

Serum concentrations of IL‐6, IL‐17A, TNF‐α, IFN‐γ, IL‐10, and SIgA were quantified using commercial ELISA kits (Beijing Dongge Biotechnology Co. Ltd., Beijing, China) according to the manufacturer's instructions. Absorbance was measured at 450 nm using a Multiskan GO microplate reader (Thermo Fisher Scientific, Waltham, MA, USA).

### 2.7 Gut Microbiota Analysis

Colonic contents were aseptically collected in accordance with Section 2.5. Four experimental groups were established, including the control group, DSS group, BF_15 group, and LGG group, with 4 biological replicates per group (Zhao et al. 2024). Total bacterial genomic DNA was extracted from mouse feces using the QIAamp Stool DNA Kit. Using the extracted DNA as the template, the V3–V4 hypervariable region (300 bp) of the bacterial 16S rDNA was amplified by PCR with universal primers 338F (5′‐ACTCCTACGGGAGGCAGCAG‐3′) and 806R (5′‐GGACTACHVGGGTWTCTAAT‐3′). All qualified PCR products were delivered to Majorbio Bio‐Pharm Technology Co. Ltd. for high‐throughput sequencing on the Illumina MiSeq PE300 platform.

Raw sequencing reads were demultiplexed and quality‐filtered using QIIME software. Operational taxonomic units (OTUs) were clustered at a 97% sequence similarity threshold via UPARSE (Version 11), and representative sequences with taxonomic information were further annotated. Subsequent bioinformatic analyses were performed on the Majorbio Cloud Platform (https://cloud.majorbio.com/), and the structural characteristics of gut microbiota were statistically analyzed at both phylum and genus levels.

### 2.8 TMT‐Based Proteomics

Colon tissues aseptically isolated in Section 2.5 were preserved on dry ice and sent to Majorbio Bio‐Pharm Technology Co. Ltd. Four experimental groups were set up as described above, with three biological replicates in each group. Whole proteomic analysis was conducted following standard experimental procedures, and the specific steps are listed below.

#### 2.8.1 Detection and Analysis of TMT Quantitative Proteomics

Frozen colon tissues were ground and fully lysed in lysis buffer supplemented with protease inhibitors. Total protein was harvested after high‐speed centrifugation. Protein concentration was quantified by the BCA method, and protein integrity was verified by SDS‐PAGE electrophoresis. Subsequently, qualified protein samples were subjected to TCEP reduction, IAM alkylation, and acetone precipitation for purification, followed by overnight trypsin digestion. Peptide fragments were labeled with TMT reagents, mixed equally, and concentrated under vacuum.

Fractionation of labeled peptides was performed by high‐performance liquid chromatography. After fraction combination, LC–MS/MS analysis was conducted to acquire raw proteomic data. Raw spectra were searched and annotated using MaxQuant software, with FDR ≤ 0.01 for proteins and peptides set as the qualitative cutoff. Differentially expressed proteins (DEPs) were screened based on the thresholds of fold change > 1.2 or < 0.83 and *p* < 0.05. Differences in protein expression profiles were compared among the normal group, DSS model group, and probiotic pretreatment groups.

#### 2.8.2 Bioinformatic Analysis of Differential Proteins

All identified DEPs were subjected to systematic bioinformatic analysis. The UPGMA algorithm was adopted for cluster analysis. With the assistance of the GeneAnswers tool and the Hypergeometric test, KEGG pathway enrichment analysis was performed based on public databases.

### 2.9 Statistical Analysis

All experimental data are expressed as mean ± standard deviation (mean ± SD). Statistical analysis was performed using the SPSS 17.0 software (IBM Corp., Armonk, NY, USA), while data processing and graph plotting were conducted using GraphPad Prism 5 software. One‐way analysis of variance (One‐way ANOVA) followed by Duncan's multiple comparison test was used for between‐group difference comparisons, with *p* < 0.05 considered a statistically significant difference.

## 3 Results

### 3.1 BF_15 Alleviated DSS‐Induced Clinical Symptoms

**FIGURE 1:**
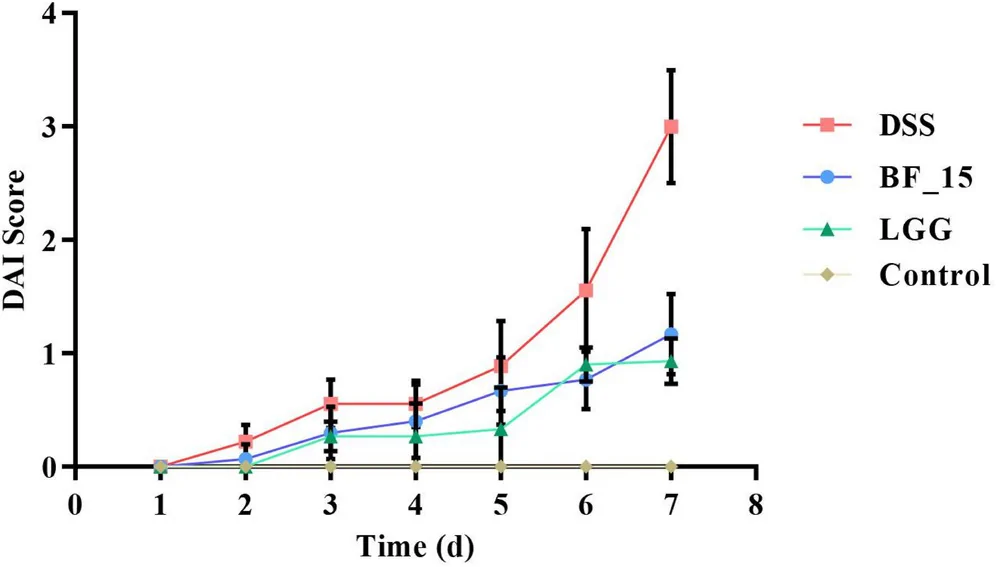
The DAI score of mice in different groups.

DAI scores, reflecting weight loss, stool consistency, and fecal blood, were markedly elevated in DSS‐treated mice compared with controls (3.00 ± 0.50 vs. ~0; *p* < 0.05). Probiotic supplementation with BF_15 or LGG significantly reduced DAI scores (1.16 ± 0.36 and 0.93 ± 0.20, *p* < 0.05), accompanied by improved activity, coat condition, and stool consistency (Figure 1). These findings indicate that BF_15 effectively mitigated DSS‐induced colitis symptoms, comparable to LGG.

### 3.2 BF_15 Improved Colonic Pathology

**FIGURE 2:**
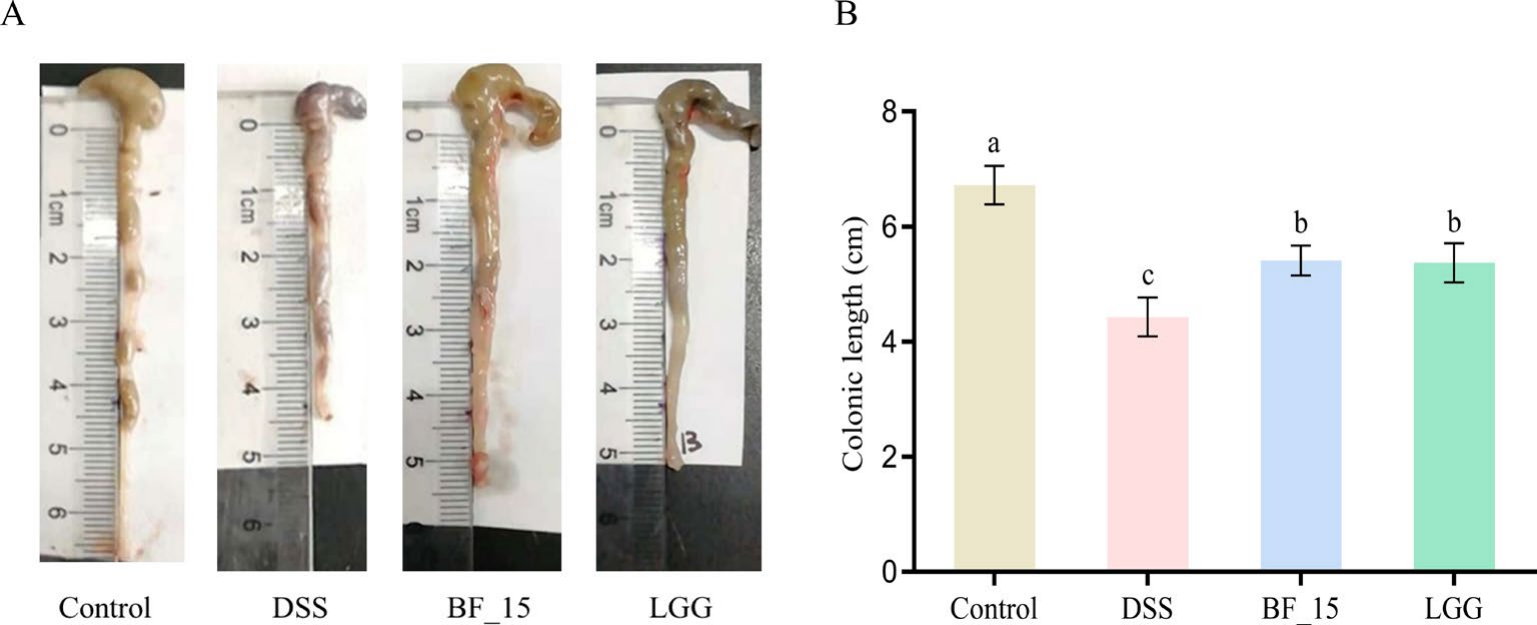
Effects of BF_15 on the physiological indexes of DSS‐induced UC mice. (A) Colon morphology of mice in different groups; (B) Colon length of mice in different groups. Results are presented as the mean ± SD (*n* = 10). The lowercase letters (a–c) indicate significant differences (*p* < 0.05) among different groups within the same experimental period.

Macroscopic examination revealed pronounced colon edema and bleeding in DSS‐treated mice, whereas probiotic treatment alleviated visible bleeding and reduced swelling (Figure 2A). Colon length was significantly shortened in the DSS group (4.43 ± 0.32 cm vs. 6.72 ± 0.32 cm in controls, *p* < 0.05). Both BF_15 and LGG partially restored colon length (5.41 ± 0.26 cm and 5.37 ± 0.34 cm, *p* < 0.05), confirming protective effects against DSS‐induced colonic injury (Figure 2B).

### 3.3 BF_15 Modulated Systemic Inflammatory Responses

**FIGURE 3:**
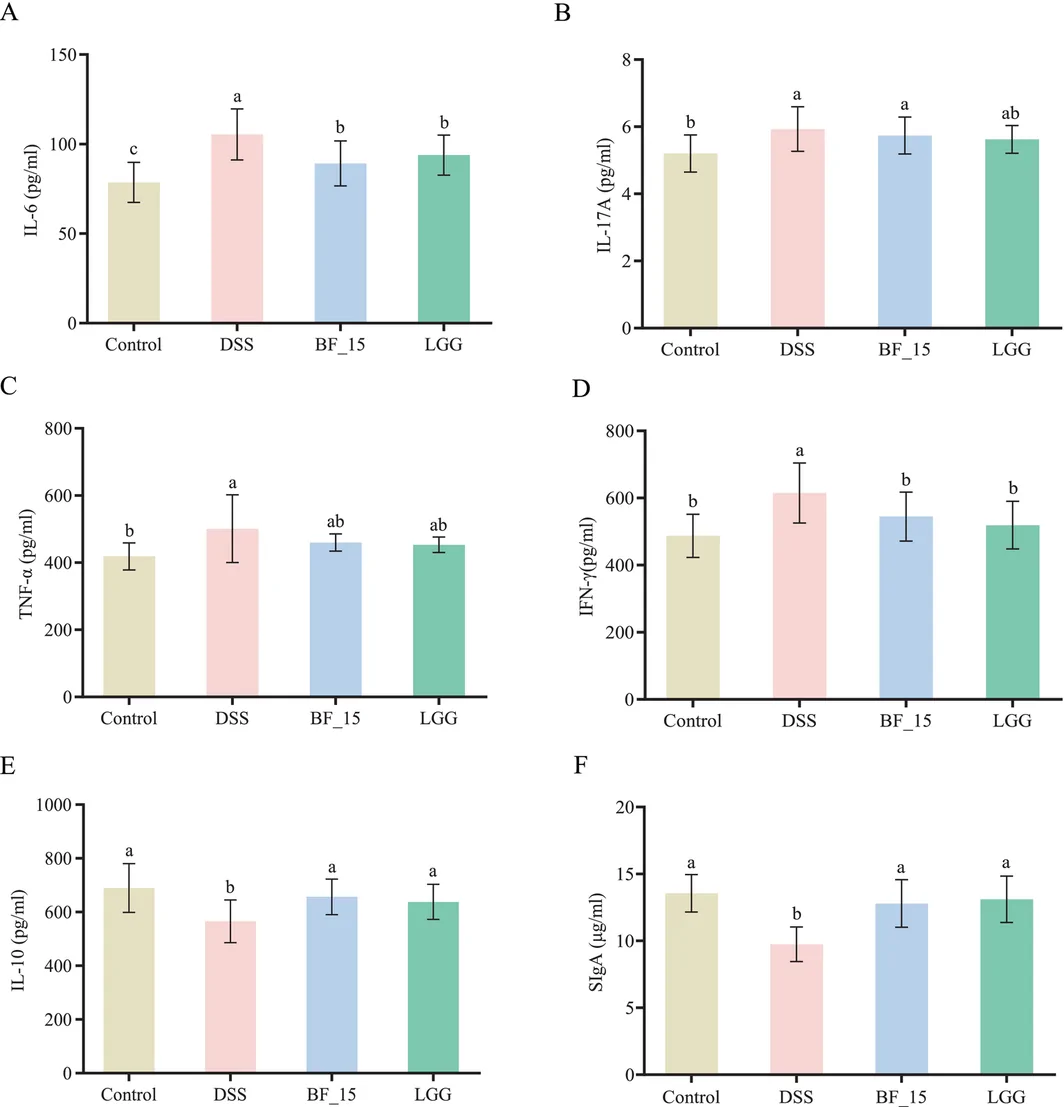
The contents of inflammatory factors (IL‐6, IL‐17A, TNF‐α, IFN‐γ, and IL‐10) and SIgA in serum. (A) IL‐6; (B) IL‐17A; (C) TNF‐α; (D) IFN‐γ; (E) IL‐10; (F) SIgA. Results are presented as the mean ± SD (*n* = 10). The lowercase letters (a–c) indicate significant differences (*p* < 0.05) among different groups within the same experimental period.

As shown in Figure 3, DSS treatment increased the levels of proinflammatory cytokines IL‐6 (105.42 ± 14.24 vs. 78.58 ± 11.18 pg/mL), IL‐17A (5.93 ± 0.66 vs. 5.20 ± 0.55 pg/mL), TNF‐α (501.17 ± 100.91 vs. 418.68 ± 40.56 pg/mL), and IFN‐γ (615.09 ± 89.25 vs. 487.47 ± 64.50 pg/mL) and decreased the levels of anti‐inflammatory cytokines IL‐10 (565.92 ± 79.40 vs. 689.43 ± 90.76 pg/mL) and SIgA (9.75 ± 1.29 vs. 14.26 ± 2.00 μg/mL) compared with the control group (*p* < 0.05). Probiotic modulation by BF_15/LGG could reverse DSS‐induced inflammatory response: The levels of IL‐6 (89.24 ± 12.60/93.88 ± 11.16 pg/mL), TNF‐α (459.69 ± 25.69/453.07 ± 22.97 pg/mL), and IFN‐γ (544.72 ± 73.00/519.24 ± 71.16 pg/mL) were close to the control group, while the level of IL‐10 (656.84 ± 66.01/638.18 ± 65.21 pg/mL) and SIgA (12.79 ± 1.77/13.11 ± 1.74 μg/mL) were also recovered (*p* < 0.05). Of note, our results also showed that BF_15 could rescue UC inflammation as efficiently as LGG.

### 3.4 BF_15 Reshaped Gut Microbiota Composition

**FIGURE 4:**
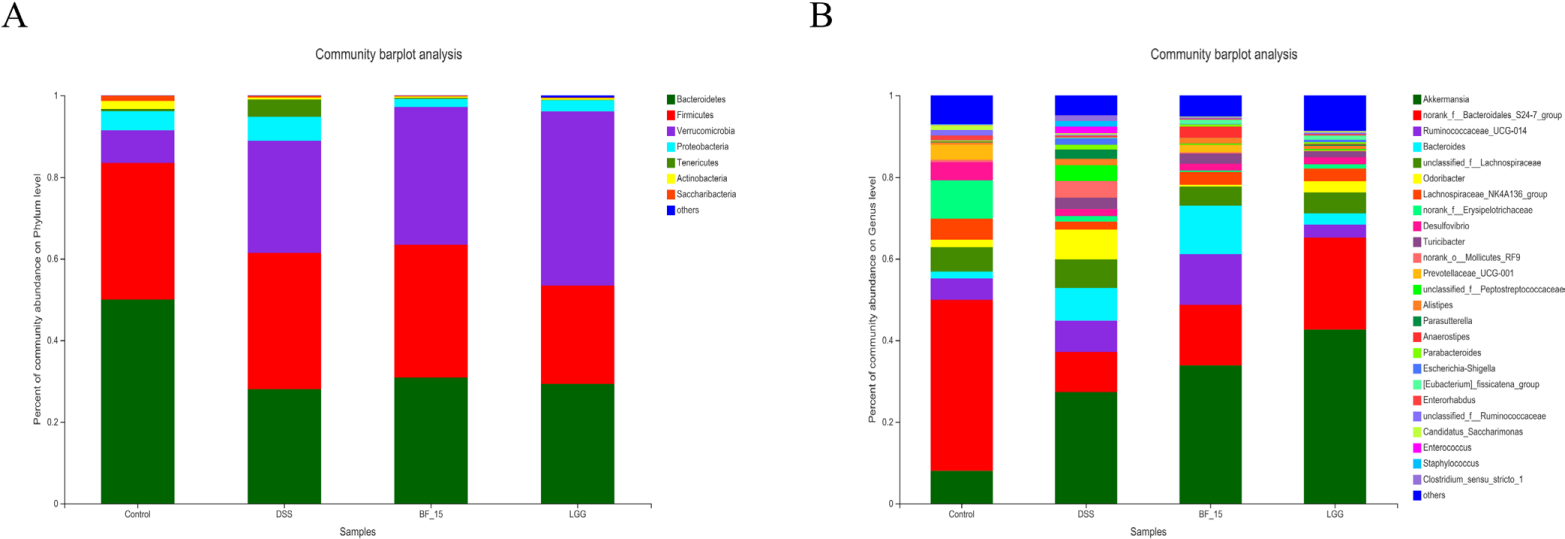
Effect of probiotics (BF_15/LGG) on intestinal flora of UC mice induced by DSS. (A) Composition of flora between different treatment groups at phylum level; (B) Composition of flora between different treatment groups at genus level.

As shown in Figure 4, compared to the normal control group, DSS treatment significantly reduced the relative abundance of *Bacteroidetes* (from 49.92% to 28.02%) and *Firmicutes* (from 33.52% to 33.48%). Concurrently, DSS administration induced an increase in the relative abundance of *Proteobacteria*, *Tenericutes*, and *Verrucomicrobia*. Notably, BF_15/LGG intervention effectively mitigated the decline in *Bacteroidetes* abundance (30.85%, 29.29%) and suppressed the abnormal proliferation of *Proteobacteria* and *Tenericutes* (Figure 4A).

At the genus level, DSS treatment (M) decreased the abundance of beneficial bacterial groups, including the *Bacteroidales* S24‐7 group, *Lachnospiraceae* NK4A136 group, and *Desulfovibrio*, while increasing the abundance of *Ruminococcaceae* UCG‐014 and *Bacteroides*. BF_15/LGG intervention partially reversed this dysbiosis, promoting the recovery of *Lachnospiraceae* and *Bacteroidales* (Figure 4B).

### 3.5 Proteomic Profiling Revealed BF_15‐Mediated APC Pathway Modulation

**FIGURE 5:**
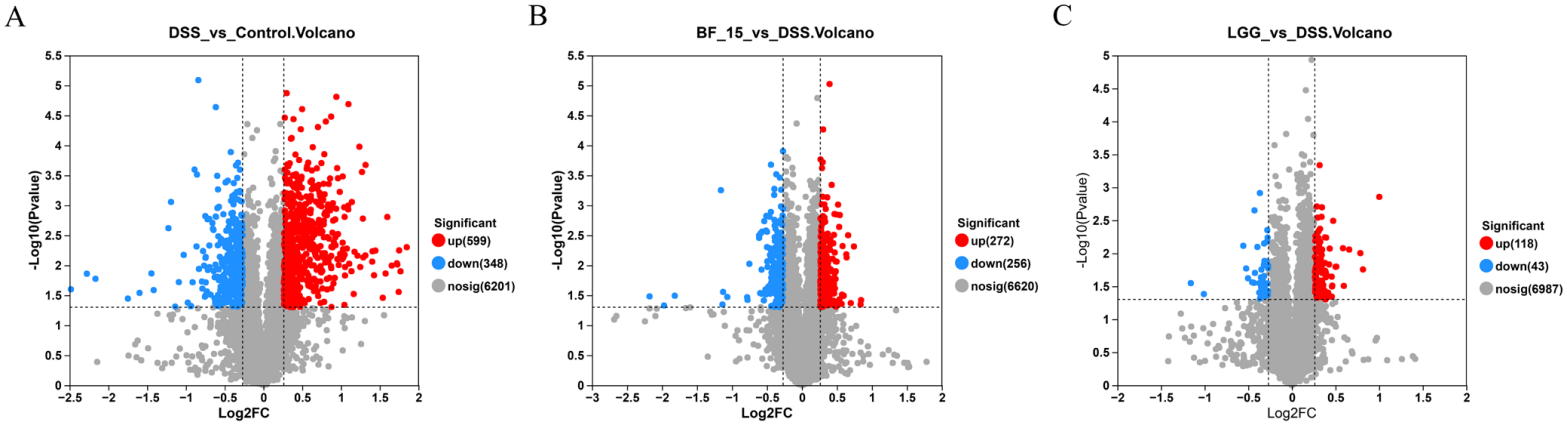
Volcano plot of differentially expressed proteins in different comparison groups: (A) Relative to the normal group, the differentially expressed proteins in the UC model group. (B) Relative to the UC model group, the differentially expressed proteins in the BF_15 group. (C) Relative to the UC model group, the differentially expressed proteins of LGG. Red represents down‐regulated and blue represents up‐regulated.

TMT‐based quantitative proteomics identified 35,531 proteins at 1% FDR, of which 947 were differentially expressed in DSS mice (599 upregulated, 348 downregulated vs. control). BF_15 treatment altered 528 proteins (272 upregulated, 256 downregulated vs. DSS), while LGG affected 161 proteins (Figure 5).

**FIGURE 6:**
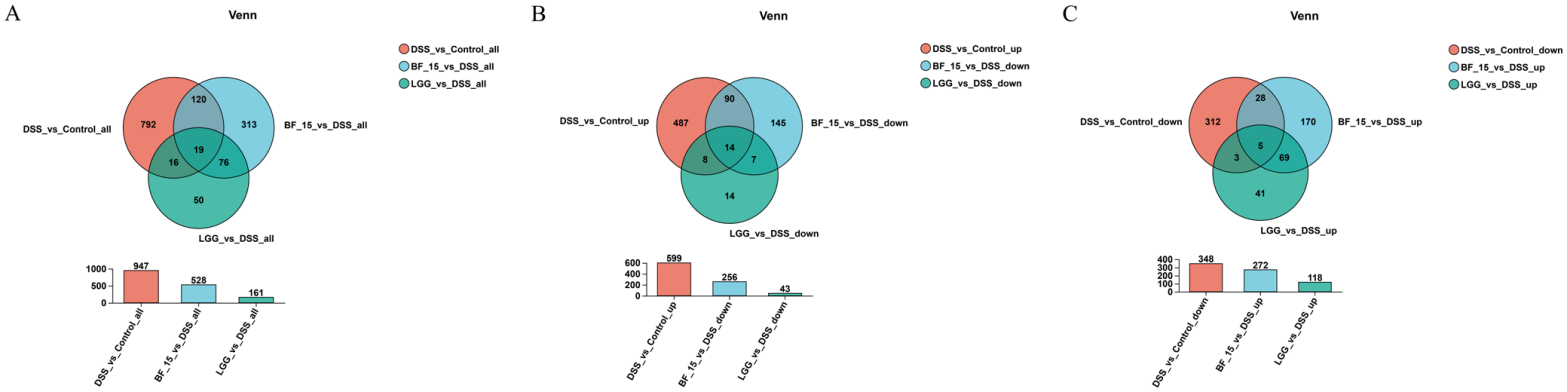
Comparative analysis of differentially expressed proteins of UC and UC intervened with probiotics (BF_15/LGG) in colon tissue. (A) All differentially expressed proteins; (B) BF_15 and LGG groups down‐regulate differentially expressed proteins; (C) BF_15 and LGG groups up‐regulate differentially expressed proteins.

Comparative analysis revealed 139 proteins uniquely reversed by BF_15 and 35 by LGG, with partial overlap (Figure 6). Key proteins regulated by BF_15 included antioxidant glutathione peroxidase (GSH‐Px) and polymeric immunoglobulin receptor (pIgR), as well as suppression of pro‐inflammatory mediators such as legumain, ALOX15, MMP‐2, and ApoC‐III (Table 2 and Table S1). KEGG enrichment highlighted significant involvement of the antigen processing and presentation (APC) pathway, with BF_15 modulating a broader set of APC‐associated proteins compared to LGG (Table 3).

**TABLE 2:** Differentially expressed proteins directly back‐regulated (up regulated) by BF_15/LGG.

Accession	Description	FC (DSS/Control)	FC (BF_15/DSS)	FC (LGG/DSS)
E9Q8Z5	Catenin delta‐1	0.779817	1.203209	――
P00375	Dihydrofolate reductase	0.829971	1.336806	――
E9PUA7	Tumor protein D52	0.793376	1.277594	――
Q544R9	High mobility group box 3	0.806108	1.251838	――
E9Q8Z8	Catenin delta‐1	0.789234	1.275145	――
P98191	Phosphatidate cytidylyltransferase 1	0.735631	1.383717	――
Q9CQD1	Ras‐related protein Rab‐5A	0.743026	1.226394	――
P59644	Phosphatidylinositol 4,5‐bisphosphate 5‐phosphatase A	0.803379	1.202683	――
Q9DBJ3	Brain‐specific angiogenesis inhibitor 1‐associated protein 2‐like protein 1	0.737252	1.263775	――
Q571M4	MKIAA4014 protein (Fragment)	0.778105	1.208223	――
A0A0R4J111	Glutathione peroxidase	0.737921	1.505152	――
F8WGU3	Epithelial‐splicing regulatory protein 1	0.82381	1.219653	――
D3YWN7	Ensconsin	0.732189	1.232122	――
P50431	Serine hydroxymethyltransferase, cytosolic	0.801212	1.209724	――
Q3UPC8	Uncharacterized protein	0.750399	1.240393	――
Q8BGB2	Tetratricopeptide repeat protein 7A	0.815207	1.225502	――
Q3TAD4	Uncharacterized protein	0.788246	1.226036	――
A0A0R4J1L9	Microtubule‐associated tumor suppressor 1 homolog	0.810756	1.244174	――
Q8BGS1	Band 4.1‐like protein 5	0.808775	1.230476	――
Q9Z1W9	STE20/SPS1‐related proline‐alanine‐rich protein kinase	0.750269	1.227756	――
Q8C0V0	Serine/threonine‐protein kinase tousled‐like 1	0.743544	1.215569	――
P97805	Protein FAM3D	0.594791	1.280375	――
Q3U4P3	Uncharacterized protein (Fragment)	0.790856	1.253383	――
Q9JLZ8	Single Ig IL‐1‐related receptor	0.707248	1.234598	――
Q0PD30	RAB25, member RAS oncogene family, isoform CRA_b	0.742662	1.229901	――
Q545J5	Basic Kruppel‐like transcription factor	0.74015	1.277567	――
Q3TYB1	Uncharacterized protein	0.668182	1.367347	――
Q8VI23	Solute carrier family 12 member 8	0.540015	1.201801	――
**Q68G78**	**Csda protein**	**0.750899**	**1.552224**	**1.300239**
**Q8CI08**	**SLAIN motif‐containing protein 2**	**0.623515**	**1.579992**	**1.266773**
**O70570**	**Polymeric immunoglobulin receptor**	**0.79822**	**1.35316**	**1.255266**
**E9QM38**	**Solute carrier family 12 member 2**	**0.72441**	**1.290242**	**1.272704**
**Q9ES88**	**Solute carrier family 13 member 2**	**0.712878**	**1.311482**	**1.375393**
P63089	Pleiotrophin	0.577008	――	1.237931
A8CA96	Tissue‐specific transcription factor S‐II	0.666695	――	1.263795
Q60829	Protein phosphatase 1 regulatory subunit 1B	0.663453	――	1.230779

*Note:* ―― represents that there is no callback effect, and the bold part of the font is the common callback part of BF**_**15 and LGG, FC > 1 represents upregulation, FC < 1 represents downregulation.

**TABLE 3:** The potential target pathway of UC intervened with probiotics (BF_15/LGG) in colon tissue.

The potential target pathway	Proteins involved				
	Accession	Description	FC (DSS/Control)	FC (BF_15/DSS)	FC (LGG/DSS)
Antigen processing and presentation	A0A068BER1	Transporter 1 ATP‐binding cassette sub‐family B	1.322489	――	――
	A0A068BIS1	TAP binding protein	1.31229	――	――
	A2RTI3	Legumain	2.490881	0.654324	0.792887
	P01887	Beta‐2‐microglobulin	1.410633	――	0.802727
	P01899	H‐2 Class I histocompatibility antigen, D‐B alpha chain	1.368691	――	――
	P01901	H‐2 Class I histocompatibility antigen, K‐B alpha chain	1.200978	――	――
	P10605	Cathepsin B	1.814286	0.812598	――
	P14429	H‐2 Class I histocompatibility antigen, Q7 alpha chain	1.410502	――	――
	P14434	H‐2 Class II histocompatibility antigen, A‐B alpha chain	1.27214	――	――
	P17156	Heat shock‐related 70 kDa protein 2	1.363586	0.780553	――
	P36371	Antigen peptide transporter 2	1.326698	――	――
	Q31093	Histocompatibility 2, M region locus 3	1.209416	――	――
	Q31106	MAd protein	1.273015	――	――
	Q3UD32	Uncharacterized protein	1.238149	――	――
	Q8HWB2	Histocompatibility 2, Q region locus 4	1.360902	――	――
	P07901	Heat shock protein HSP 90‐alpha	――	1.217186	――
	Q71LX8	Heat shock protein 84b	――	1.445882	1.208235

*Note:* ―― represents that there is no callback effect, FC > 1 represents upregulation, FC < 1 represents downregulation.

## 4 Discussion

Compared with probiotics derived from traditional fermented products, human‐derived probiotics (especially mother‐infant‐derived strains) exhibit more prominent advantages in terms of human host biocompatibility, application safety, functional specificity, and clinical translational potential (Yang et al. 2018). The results of this study confirm that infant‐derived *L. plantarum* BF_15 can effectively alleviate DSS‐induced murine colitis, with an intervention effect comparable to that of the well‐recognized reference strain LGG. This work provides a novel candidate strain for probiotic intervention strategies against UC.

The imbalance between pro‐inflammatory and anti‐inflammatory cytokines and the disturbance of sIgA homeostasis are important triggers for the development of UC (Neurath 2024). The results of this study confirmed that BF_15 intervention effectively reduced the DAI, alleviated body weight loss, improved systemic inflammatory status in mice with DSS‐induced colitis, significantly downregulated the secretion of pro‐inflammatory factors such as IL‐6 and IL‐17A, and simultaneously increased the levels of IL‐10 and sIgA, with an overall intervention effect comparable to that of the classic probiotic LGG. Multiple studies have confirmed that probiotics can alleviate UC injury by regulating the balance of inflammatory factors. *Limosilactobacillus fermentum* GLF‐217 can inhibit the expression of multiple pro‐inflammatory factors, upregulate tight junction and mucin‐related proteins, and repair the intestinal mucosa (Li et al. 2024). Fermented squid‐derived *L. plantarum* HY7718 can also alleviate colitis symptoms by reducing inflammatory levels and ameliorating pathological damage (Kim et al. 2024). In comparison to the above strains, the infant‐derived BF_15 used in this study not only possesses the same immunomodulatory capacity, but its infant‐derived origin also endows it with stronger host immune biocompatibility and intestinal colonization advantages, highlighting the original value of this study and the application potential of BF_15 (Zhang et al. 2020).

Gut microbiota analysis showed that early intervention with BF_15 effectively reversed the DSS‐induced decrease in the abundance of Bacteroidetes and Firmicutes and suppressed the aberrant expansion of Proteobacteria, thereby ameliorating gut microecological dysbiosis and alleviating colonic inflammatory injury. Multiple studies have confirmed that probiotics alleviate UC lesions by targeted regulation of microbiota structure: *Lactobacillus helveticus* LZ‐R‐5 can inhibit the excessive proliferation of potential harmful bacteria, promote the enrichment of beneficial bacteria such as *Muribaculaceae* and *Lachnospiraceae*, and maintain microbiota homeostasis (Zhao et al. 2025). *Lactiplantibacillus plantarum* HY7718 reverses DSS‐mediated microbiota dysbiosis by downregulating the abundance of Proteobacteria and upregulating the abundance of Actinobacteria (Kim et al. 2024). Taken together, different probiotics can exert anti‐inflammatory and protective effects by regulating gut microbiota composition, but there are strain‐specific differences in the action targets and the regulated bacterial phyla.

TMT‐based quantitative proteomics results confirmed that the APC pathway is the core pathway for BF_15 to regulate UC. Compared with the reference strain LGG, BF_15 can regulate a wider range of APC‐associated proteins, reflecting its unique intervention advantage. In a study on the amelioration of UC by 7,8‐dihydroxyflavone (7,8‐DHF), transcriptomic analysis revealed that 7,8‐DHF can ameliorate UC by reversing the aberrant expression of colonic inflammation‐related genes induced by DSS and further inhibiting inflammatory pathways including the APC pathway (Wang et al. 2026). To date, few studies have reported that probiotics exert UC‐ameliorating effects by targeting the APC pathway in existing research on probiotic intervention for UC. The results of this study fill this research gap and enrich the research on the molecular mechanism of probiotic intervention in UC.

## 5 Conclusion

Combining in vivo animal experiments, 16S rRNA gene sequencing, and TMT‐based quantitative proteomics, this study provides the first confirmed demonstration that breastfed infant‐derived *L. plantarum* BF_15 ameliorates UC via regulating the APC pathway and restoring gut microbiota homeostasis. This study provides novel insights into the targeted probiotic intervention of UC. It should be noted that the protective effect of BF_15 was only validated in a murine model in this study, and its efficacy remains to be further verified in clinical trials.

## Author Contributions

**Hongjie Liu:** writing – review and editing, validation, data curation. **Na Zhang:** conceptualization, methodology, software, investigation, data curation, formal analysis, visualization, writing – original draft. **Hongtao Tian:** conceptualization, methodology, supervision, funding acquisition. **Xiaoyan Miao:** visualization, data curation. **Xinyi Ge:** validation, data curation. **Dongyao Li:** conceptualization, software, funding acquisition, writing – review and editing. **Chen Li:** software, writing – review and editing. **Miaoshu Wang:** data curation, visualization. **Hongyan Kang:** data curation, validation.

## Conflicts of Interest

Co‐authors Hongyan Kang and Miaoshu Wang are employed by New Hope Tensun (Hebei) Dairy Co. Ltd. and act as off‐campus supervisors of the affiliated university. They provided auxiliary technical support for this study based on industrial practical experience, including experimental validation, data curation, and figure visualization. Their affiliated company did not provide any financial support, experimental supplies, or laboratory resources for this study. The patent of strain BF_15 (No. ZL202010193432.4) is exclusively owned by the university of the corresponding institution. No commercial development or industrial translation of strain BF_15 has been conducted so far. The other authors declare no conflicts of interest.

## Supporting information


**Figure S1:** Schematic illustration of animal experimental design. All mice were maintained under SPF conditions with a 7‐day acclimatization period, then randomly allocated into four groups (*n* = 10 per group). The Control group received daily oral gavage of 0.9% saline throughout the experiment. The DSS group was intragastrically administered saline followed by DSS solution. The BF_15 and LGG groups were given daily oral gavage of *Lactiplantibacillus plantarum* BF_15 and LGG suspension (1 × 10^9^ CFU/mL, 0.1 mL/10 g body weight), respectively, for 3 weeks. Starting at Week 4, mice from the DSS, BF_15 and LGG groups were orally treated with 0.8 g/mL DSS solution (0.1 mL/10 g body weight) for 7 consecutive days to induce acute ulcerative colitis.
**Table S1:** Differentially expressed proteins directly back‐regulated (down regulated) by BF_15/LGG.

## Data Availability

The data that support the findings of this study are available from the corresponding author upon reasonable request.
