# Supplementary material for: Lactiplantibacillus plantarum BF_15 Confers Protection Against Ulcerative Colitis by Orchestrating Gut Microbiota‐Barrier‐Immune Triad Homeostasis
Source: Food Sci Nutr. 2026 Sep 4;14(9):e72275. doi: 10.1002/fsn3.72275 (PMC13545421; doi:10.1002/fsn3.72275)
Supplement: Supplementary file 1 [file FSN3-14-e72275-s001.docx]

**Figure S1 Schematic illustration of animal experimental design.**

All mice were maintained under SPF conditions with a 7-day acclimatization period, then randomly allocated into four groups (n = 10 per group). The Control group received daily oral gavage of 0.9% saline throughout the experiment. The DSS group was intragastrically administered saline followed by DSS solution. The BF_15 and LGG groups were given daily oral gavage of *Lactiplantibacillus plantarum* BF_15 and LGG suspension (1 × 10⁹ CFU/mL, 0.1 mL/10 g body weight), respectively, for 3 weeks. Starting at week 4, mice from the DSS, BF_15 and LGG groups were orally treated with 0.8 g/mL DSS solution (0.1 mL/10 g body weight) for 7 consecutive days to induce acute ulcerative colitis.

**Table S1** Differentially expressed proteins directly back-regulated (down regulated) by BF_15/LGG

| Accession | Description | FC(M/CK) | FC(BF_15/M) | FC(LGG/M) |
| --- | --- | --- | --- | --- |
| A0A075DCG2 | Cytochrome c oxidase subunit 3 | 1.286927 | 0.726913 | ―― |
| A0A0R4J1E2 | Elongation factor 1-delta | 1.522246 | 0.755045 | ―― |
| Q00623 | Apolipoprotein A-I | 2.643813 | 0.581278 | ―― |
| E9PVD3 | Protocadherin-16 | 1.431146 | 0.80963 | ―― |
| Q61409 | cGMP-inhibited 3',5'-cyclic phosphodiesterase B | 1.235462 | 0.814043 | ―― |
| P10605 | Cathepsin B | 1.814286 | 0.812598 | ―― |
| A0A0N4SUM4 | Neural cell adhesion molecule L1-like protein | 1.509258 | 0.771373 | ―― |
| A0A0R4J0K5 | SLAM family member 5 | 1.833026 | 0.744617 | ―― |
| P34928 | Apolipoprotein C-I | 1.977836 | 0.751984 | ―― |
| Q3U8W3 | Uncharacterized protein | 1.469989 | 0.819886 | ―― |
| Q61549 | Adhesion G protein-coupled receptor E1 | 1.540984 | 0.824877 | ―― |
| Q3T9B7 | Phospholipid scramblase | 1.337717 | 0.707373 | ―― |
| P61028 | Ras-related protein Rab-8B | 1.43758 | 0.801543 | ―― |
| Q9R0X4 | Acyl-coenzyme A thioesterase 9, mitochondrial | 1.309322 | 0.787217 | ―― |
| Q3UFA6 | Uncharacterized protein | 1.655283 | 0.78665 | ―― |
| Q2HZ94 | Macrophage mannose receptor 1 | 1.716709 | 0.723922 | ―― |
| Q3U2S8 | Voltage-gated hydrogen channel 1 | 1.608079 | 0.746423 | ―― |
| P17156 | Heat shock-related 70 kDa protein 2 | 1.363586 | 0.780553 | ―― |
| Q8BTD8 | Uncharacterized protein (Fragment) | 1.452409 | 0.770469 | ―― |
| P48036 | Annexin A5 | 1.3732 | 0.763468 | ―― |
| Q3TFI6 | GTPase IMAP family member 1 | 1.57335 | 0.698757 | ―― |
| A0A0B4J1G1 | Fc receptor, IgG, low affinity IIb | 1.900355 | 0.772159 | ―― |
| P16045 | Galectin-1 | 1.379506 | 0.793322 | ―― |
| Q9DCJ9 | N-acetylneuraminate lyase | 1.51519 | 0.823574 | ―― |
| Q8CGA3 | Large neutral amino acids transporter small subunit 4 | 1.712291 | 0.77137 | ―― |
| Q6ZWR0 | MCG5466 | 1.564246 | 0.803156 | ―― |
| Q9R1Q6 | Transmembrane protein 176B | 1.592161 | 0.746018 | ―― |
| Q6PKB0 | Fcgrt protein | 1.382483 | 0.740176 | ―― |
| E9QPX1 | Collagen alpha-1(XVIII) chain | 1.622887 | 0.726522 | ―― |
| Q6ZPJ3 | (E3-independent) E2 ubiquitin-conjugating enzyme UBE2O | 1.28684 | 0.806208 | ―― |
| A2VCP3 | P2X purinoceptor (Fragment) | 1.482121 | 0.784276 | ―― |
| P70170 | ATP-binding cassette sub-family C member 9 | 1.58816 | 0.729335 | ―― |
| P50285 | Dimethylaniline monooxygenase [N-oxide-forming] 1 | 1.300867 | 0.798482 | ―― |
| P03995 | Glial fibrillary acidic protein | 1.537502 | 0.727841 | ―― |
| Q8K4Q8 | Collectin-12 | 1.434324 | 0.757225 | ―― |
| Q923B6 | Metalloreductase STEAP4 | 1.473052 | 0.804499 | ―― |
| Q60648 | Ganglioside GM2 activator | 1.329599 | 0.826569 | ―― |
| Q6NZL0 | Protein SOGA3 | 1.306615 | 0.765004 | ―― |
| Q3UIA4 | Aldehyde dehydrogenase | 1.511719 | 0.828596 | ―― |
| P14602 | Heat shock protein beta-1 | 1.249338 | 0.796438 | ―― |
| B9EKB0 | Laminin B1 subunit 1 | 1.299655 | 0.8125 | ―― |
| Q8CDV7 | Ectonucleoside triphosphate diphosphohydrolase 1 | 1.394617 | 0.765497 | ―― |
| P08074 | Carbonyl reductase [NADPH] 2 | 1.264703 | 0.802901 | ―― |
| Q62048 | Astrocytic phosphoprotein PEA-15 | 1.237954 | 0.807528 | ―― |
| E9Q8B5 | Predicted gene 4788 | 1.431563 | 0.816954 | ―― |
| A0A0R4J097 | Transforming growth factor beta receptor type 3 | 1.692162 | 0.6856 | ―― |
| Q3U254 | Uncharacterized protein | 1.33979 | 0.813253 | ―― |
| Q3UGJ5 | Uncharacterized protein | 1.468833 | 0.814434 | ―― |
| P62737 | Actin, aortic smooth muscle | 1.230612 | 0.735738 | ―― |
| Q8BFW6 | Ectonucleoside triphosphate diphosphohydrolase 3 | 1.314544 | 0.752931 | ―― |
| Q3TDQ8 | Retinal rod rhodopsin-sensitive cGMP 3',5'-cyclic phosphodiesterase subunit delta | 1.243674 | 0.810649 | ―― |
| Q64449 | C-type mannose receptor 2 | 1.528007 | 0.735079 | ―― |
| O54865 | Guanylate cyclase soluble subunit beta-1 | 1.227795 | 0.775574 | ―― |
| Q61553 | Fascin | 1.4 | 0.804329 | ―― |
| P70232 | Neural cell adhesion molecule L1-like protein | 1.306215 | 0.802509 | ―― |
| Q9QZF2 | Glypican-1 | 1.348328 | 0.780411 | ―― |
| P18872 | Guanine nucleotide-binding protein G(o) subunit alpha | 1.203886 | 0.78515 | ―― |
| Q8K0E9 | Dhdh protein (Fragment) | 1.286652 | 0.76301 | ―― |
| B1ARB3 | Platelet endothelial cell adhesion molecule | 1.278085 | 0.828004 | ―― |
| Q8R138 | Transmembrane protein 119 | 1.268356 | 0.773328 | ―― |
| Q3UY68 | Calcium/calmodulin-dependent protein kinase I | 1.349412 | 0.802354 | ―― |
| Q62277 | Synaptophysin | 1.217558 | 0.813158 | ―― |
| G5E846 | Peripherin | 1.231612 | 0.800959 | ―― |
| Q99JR5 | Tubulointerstitial nephritis antigen-like | 1.209014 | 0.823057 | ―― |
| P63213 | Guanine nucleotide-binding protein G(I)/G(S)/G(O) subunit gamma-2 | 1.300443 | 0.80271 | ―― |
| Q6ZWS7 | Uncharacterized protein | 1.392479 | 0.738143 | ―― |
| O88322 | Nidogen-2 | 1.265388 | 0.802542 | ―― |
| E9QPU1 | von Willebrand factor | 1.271657 | 0.761525 | ―― |
| Q3V0T8 | Uncharacterized protein | 1.2213 | 0.812621 | ―― |
| E9Q450 | Tropomyosin alpha-1 chain | 1.35846 | 0.772688 | ―― |
| P51637 | Caveolin-3 | 1.216561 | 0.789703 | ―― |
| Q9D1H9 | Microfibril-associated glycoprotein 4 | 1.257294 | 0.791331 | ―― |
| A2AN96 | Retinoblastoma binding protein 9 | 1.234816 | 0.74876 | ―― |
| O55186 | CD59A glycoprotein | 1.26311 | 0.821429 | ―― |
| A0A3B0J1H7 | Adiponectin d | 1.23125 | 0.725635 | ―― |
| A0A0G2JEX1 | Nexilin | 1.256287 | 0.813167 | ―― |
| P82343 | N-acylglucosamine 2-epimerase | 1.412798 | 0.808792 | ―― |
| Q3U125 | Family with sequence similarity 213, member A | 1.290037 | 0.747423 | ―― |
| Q64151 | Semaphorin-4C | 1.279664 | 0.798314 | ―― |
| O70423 | Membrane primary amine oxidase | 1.229623 | 0.777261 | ―― |
| P70274 | Selenoprotein P | 1.428031 | 0.787986 | ―― |
| F8WGF2 | Nitric oxide synthase, brain | 1.238476 | 0.813184 | ―― |
| P14824 | Annexin A6 | 1.362424 | 0.79742 | ―― |
| P24529 | Tyrosine 3-monooxygenase | 1.33774 | 0.808356 | ―― |
| P62631 | Elongation factor 1-alpha 2 | 1.200385 | 0.790553 | ―― |
| Q6PAM0 | 5'-AMP-activated protein kinase subunit beta-2 | 1.212089 | 0.752151 | ―― |
| Q8K479 | Complement C1q tumor necrosis factor-related protein 5 | 1.419266 | 0.757546 | ―― |
| P22437 | Prostaglandin G/H synthase 1 | 1.229277 | 0.821376 | ―― |
| Q14BH8 | Cacna2d1 protein | 1.353684 | 0.732255 | ―― |
| Q8CFT3 | Nerve growth factor receptor (TNFR superfamily, member 16) | 1.214115 | 0.816453 | ―― |
| **A2AVZ9** | **Solute carrier family 43 member 3** | **1.945796** | **0.700398** | **0.771461** |
| **J3QPR7** | **C-type lectin domain family 10 member A** | **2.357473** | **0.593922** | **0.801961** |
| **Q61878** | **Bone marrow proteoglycan** | **2.137715** | **0.783309** | **0.827233** |
| **A0A075B5P8** | **Immunoglobulin heavy variable 2-2 (Fragment)** | **1.837597** | **0.457809** | **0.496792** |
| **A2RTI3** | **Legumain** | **2.490881** | **0.654324** | **0.792887** |
| **Q9DCK3** | **Tetraspanin-4** | **1.947872** | **0.670463** | **0.825623** |
| **P29699** | **Alpha-2-HS-glycoprotein** | **1.797333** | **0.737908** | **0.739614** |
| **A2CF88** | **Arachidonate 15-lipoxygenase** | **1.693177** | **0.805124** | **0.783385** |
| **P97425** | **Eosinophil cationic protein 2** | **2.030942** | **0.709261** | **0.760269** |
| **Q3UG07** | **Matrix metallopeptidase 2** | **1.781079** | **0.653644** | **0.80015** |
| **E9QP56** | **Apolipoprotein C-III** | **1.831461** | **0.67408** | **0.775307** |
| **A0A140T8J4** | **Heme-binding protein 1** | **1.792545** | **0.66433** | **0.786604** |
| **Q8K2Y9** | **Cerebral cavernous malformations protein 2 homolog** | **1.266983** | **0.788405** | **0.801831** |
| **A0A0A6YXN4** | **Immunoglobulin heavy variable V1-18 (Fragment)** | **1.430658** | **0.448067** | **0.784829** |
| P07309 | Transthyretin | 1.298263 | ―― | 0.705576 |
| P40936 | Indolethylamine N-methyltransferase | 1.33035 | ―― | 0.790566 |
| P01887 | Beta-2-microglobulin | 1.410633 | ―― | 0.802727 |
| Q9Z183 | Protein-arginine deiminase type-4 | 1.86099 | ―― | 0.804545 |
| Q3UY25 | Uncharacterized protein | 2.130841 | ―― | 0.74269 |
| Q80YQ1 | Thrombospondin-1 | 1.77557 | ―― | 0.806935 |
| Q80YX1 | Tenascin | 1.542153 | ―― | 0.828175 |
| Q3TJX0 | Uncharacterized protein | 1.727145 | ―― | 0.81759 |

Note: ―― represents that there is no callback effect, and the bold part of the font is the common callback part of BF_ 15 and LGG.
